# Photocatalytic Activity of TiO_2_ Coatings Obtained at Room Temperature on a Polymethyl Methacrylate Substrate

**DOI:** 10.3390/ijms232112936

**Published:** 2022-10-26

**Authors:** Mairis Iesalnieks, Raivis Eglītis, Tālis Juhna, Krišjānis Šmits, Andris Šutka

**Affiliations:** 1 Institute of Materials and Surface Engineering, Faculty of Materials Science and Applied Chemistry, Riga Technical University, P. Valdena Street 3/7, LV1048 Riga, Latvia; 2 Water Research and Environmental Biotechnology Laboratory, Faculty of Civil Engineering, Riga Technical University, Kipsalas Street 6a, LV1048 Riga, Latvia; 3 Institute of Solid-State Physics, University of Latvia, Kengaraga Street 8, LV1063 Riga, Latvia

**Keywords:** photocatalysis, titanium dioxide, thin film

## Abstract

Titanium dioxide (TiO_2_) coatings have a wide range of applications. Anatase exhibits hydrophilic, antimicrobial, and photocatalytic properties for the degradation of organic pollutants or water splitting. The main challenge is to obtain durable anatase nanoparticle coatings on plastic substrates by using straightforward approaches. In the present study, we revealed the preparation of a transparent TiO_2_ coating on polymethylmethacrylate (PMMA), widely used for organic optical fibres as well as other polymer substrates such as polypropylene (PP), polystyrene (PS), and polycarbonate (PC). The films were spin-coated at room temperature without annealing; therefore, our approach can be used for thermo-sensitive substrates. The deposition was successful due to the use of stripped ultra-small (<4 nm) TiO_2_ particles. Coatings were studied for the photocatalytic degradation of organic pollutants such as MB, methyl orange (MO), and rhodamine B (RB) under UV light. The TiO_2_ coating on PMMA degraded over 80% of RB in 300 min under a 365 nm, 100 W mercury lamp, showing a degradation rate constant of 6 × 10^−3^ min^−1^. The coatings were stable and showed no significant decrease in degradation activity even after five cycles.

## 1. Introduction

TiO_2_ is well-known to exhibit photocatalytic properties for application in antibacterial [1] and self-cleaning surfaces [2], degradation of organic pollution [3], or water splitting [4]. The TiO_2_ coatings have been produced by different methods such as chemical vapour deposition (CVD) [5], dip-coating [6] and ultrasonic spray pyrolysis [7]. All of these approaches in most cases require annealing; thus, the substrates are limited to brittle inorganics. Organic substrates are more desired because they are flexible and thus can be applied to a wider range of applications.

TiO_2_ coatings on organic substrates have been realised by Nagasawa et al. [8], Shahmohammadi et al. [9], and Phuinthiang et al. [10]. Nagasawa et al. deposited a coating on a PMMA polymer by using atmospheric-pressure plasma-enhanced chemical vapour deposition (AP-PECVD). By using this method, Nagasawa et al. were able to produce UV-shielding TiO_2_ coatings with a decrease in UV light absorption by 99% in the 200–280 nm range and with a visible light transmittance above 95%. Although the produced coatings showed good light transmittance properties, the used method requires specific equipment and cannot be used for large surface area samples. Scanning electron microscope (SEM) images for the produced coatings showed a large agglomerate presence (larger than 300 nm) [8].

Shahmohammadi et al. used the low-temperature atomic layer deposition (ALD) method to produce a TiO_2_ coating on PMMA for use in biomedical systems. The produced coatings increased the wettability and nanohardness of the PMMA substrate but no significant changes in surface roughness were observed. The produced TiO_2_ nanocoating was able to protect the PMMA surface against thermal and brushing tests, maintaining surface integrity and wettability. The TiO_2_ film reduced the initial fungal adhesion, which can lead to the use of PMMA in biomedical applications. However, ALD is associated with high production costs and slow manufacturing speeds; in this case, to obtain a 50–80 nm thick coating, cycles must be repeated 50 times, which takes approx. 30 min [9].

Phuinthiang et al. studied the TiO_2_ coating on the polyvinylchloride (PVC) substrate. They used sol–gel and photon assistance to avoid thermal processes in thin film crystallisation. As stated in their work, they managed to obtain high-quality samples at room temperature. Produced samples showed high photocatalytic activity based on the bacteria viability assay. Phuinthiang et al. managed to produce a high-quality, low-cost coating that is easily manufacturable. Although the produced samples showed high photocatalytic activity, there are no studies regarding the repeatability of these coatings and their endurance [10].

We hypothesise that the transparent, durable, and photocatalytically active TiO_2_ coatings can be obtained from stripped ultra-small nanoparticles in a single step at room temperature. The PMMA substrate has been chosen because this polymer can be used to produce flexible optical fibres for photocatalysis reactors [11]. PMMA are flexible and cheaper than brittle quartz optical fibres.

## 2. Results

**Figure 1:**
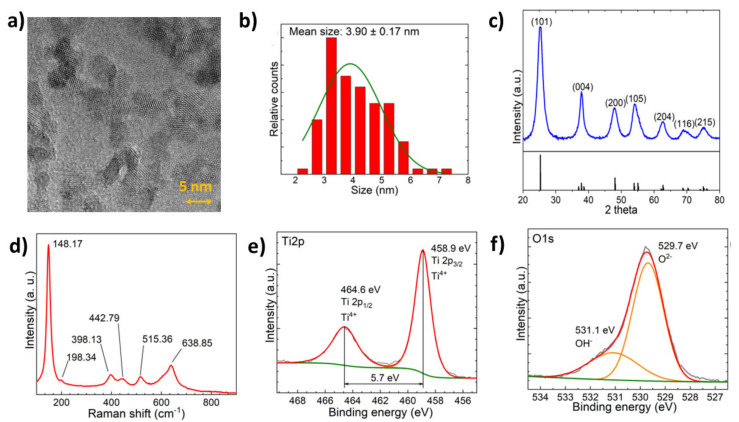
(**a**) TEM micrograph of the synthesised nanoparticles at 450,000× magnification. (**b**) Size distribution histogram for synthesised nanoparticles. (**c**) XRD diffractogram with JCPDS 21-1272 XRD data. (**d**) Raman spectra of TiO_2_ nanoparticles. (**e**) High-resolution XPS of the Ti 2p peak with peak fitting. (**f**) High-resolution XPS of the O 1s peak with peak fitting.

The performed synthesis method yielded ultra-small anatase TiO_2_ nanoparticles, as indicated by X-ray diffraction (XRD), Raman, and transmission electron microscopy (TEM) studies. The TEM images in Figure 1a revealed single-crystalline particles with the mean particle size of 3.90 nm and a particle size distribution of 0.17 nm. The anatase structure was confirmed by XRD diffractogram (Figure 1c) and the Raman spectra (Figure 1d). Both methods did not show any indication of impurity phases. In accordance with XRD, the particles consisted of an anatase phase (JCPDS 21 1272). The Raman spectra in Figure 1d showed peaks at 148.17 cm^−1^ (Eg), 198.34 cm^−1^ (Eg), 398.13 cm^−1^ (B1g), 515.36 cm^−1^ (A1g), and 6638.85 cm^−1^ (Eg), which corresponded to the anatase TiO_2_ vibrational modes [12,13]. When compared to the literature, the Raman spectra had a slight Raman band shift due to the deformation of the scattering paths caused by the small size of the particles (Figure 1d) [14,15].

The X-ray photoelectron spectroscopy (XPS) spectra of Ti 2p and O 1s are demonstrated in Figure 1e,f, respectively. The binding energy of the Ti 2p_3/2_ peak at 458.9 eV only indicates the Ti^4+^ oxidation state without shoulder peaks related to Ti^3+^. Peak splitting between Ti 2p_3/2_ and Ti 2p_1/2_ peaks was 5.7 eV, which corresponds to the literature values for anatase [16]. Oxide lattice oxygen component was found as a majority of the signal with a binding energy of 529.7 eV, while a ~26 (±1) % –OH surface hydroxyl component was present at ~531.1 eV binding energy. The signal can be interpreted as the first couple of monolayers of surface hydroxyl [17]. Band gap energy according to diffuse reflectance spectroscopy (DRS) was determined using the Tauc plot to be ~3.13 eV (Figure S1).

**Figure 2:**
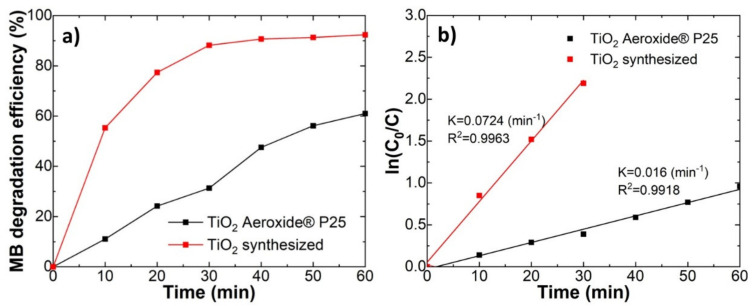
Comparison between the synthesised TiO_2_ and commercial Aeroxide ^®®^ P25 TiO_2_ powders. (**a**) the degradation efficiency of methylene blue (MB); (**b**) and the linear kinetic curves.

The synthesised TiO_2_ nanoparticles showed high photocatalytic activity. The activity for photocatalytic MB degradation (5 ppm in water) was tested for the synthesised TiO_2_ nanoparticle powders and compared with commercial Aeroxide ^®®^ P25. Figure 2a shows the MB degradation efficiency (%) for the synthesised and commercial TiO_2_. The synthesised TiO_2_ degraded around 55% MB already in 10 min, while commercial TiO_2_ can degrade only 10% at the same timescale. The synthesised TiO_2_ particles showed a 4.5-fold increase in the MB degradation rate constant (Figure 2b). The higher photocatalytic activity for synthesised TiO_2_ is due to its smaller nanoparticle size. The synthesised TiO_2_ nanoparticles had a six times smaller diameter than commercial Aeroxide ^®®^ P25 (3.9 and 25 nm, respectively).

**Figure 3:**
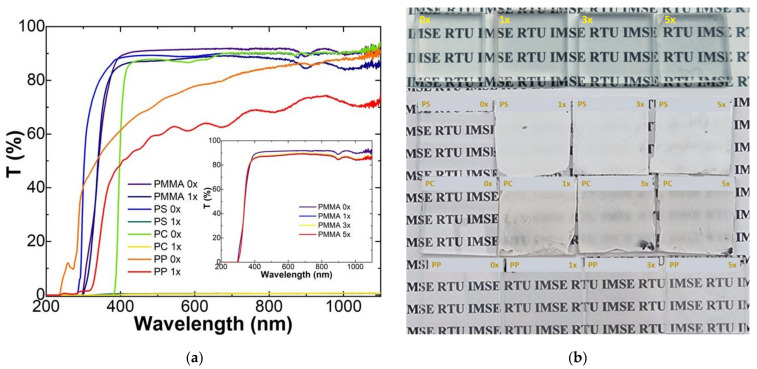
Optical properties of the produced samples. (**a**) Light transmittance changes before (0×) and after (1–5×) deposition of the TiO_2_ coating; (**b**) produced sample photography showing their translucency.

The nanoparticle coatings were deposited on different polymer substrates such as PMMA, PP, (PS), or PC. Transparent coatings without a significant decrease in transparency could be observed in the case of PMMA. Light transmission measurements showed that the light transmittance of PMMA after the TiO_2_ layer deposition did not change significantly. The PMMA samples after TiO_2_ deposition kept 97% of their initial light transmittance (Figure 3). The deposition of TiO_2_ coatings on the PS, PC, and PP substrates showed a drop in the light transmittance due to the formation of porous structures, as discussed below. A decrease in transmittance was due to light scattering by porous structures. The introduction of the TiO_2_ coating showed a slight decrease in the contact angle values for deionised water in comparison with the uncoated PMMA substrate (Figure S2).

**Figure 4:**
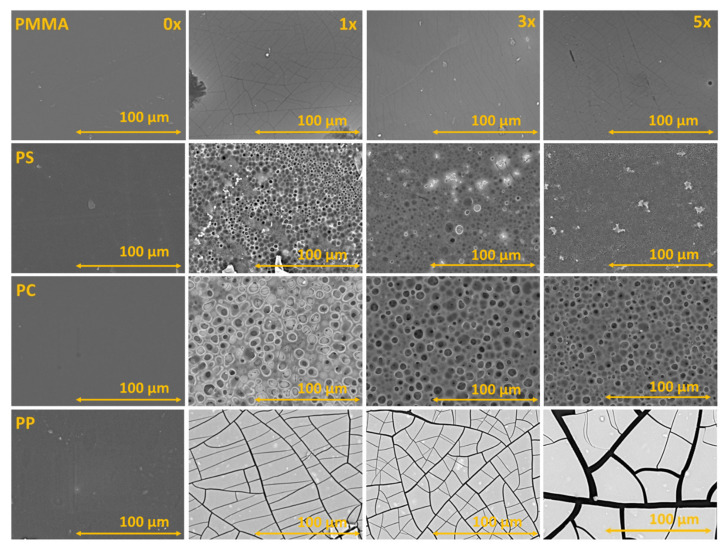
SEM images of the substrates before coating and with one, three, and five photocatalyst layers.

The SEM images of coatings on different polymer substrates are presented in Figure 4. The produced TiO_2_ coating on PMMA was homogenous and defect-free. It is worth noting that crack formation is due to the electron beam effect on the surface and is not connected with the coating properties. During SEM imaging, it was observed that cracks became bigger and more prevalent, with an increase in voltage. Before imaging, a 2.2 nm thick Au nanolayer was deposited on the coating surface to avoid surface charging and decrease the electron beam damage. Polypropylene samples showed low coating adhesion with the surface as shown in Figure 4. Large crack formation can be observed, especially with coatings with a larger number of layers. In the case of PS and PC, the formation of pores can be observed that could be related to solvent–polymer interaction. The pores are responsible for turning the substrate white and non-transparent due to light scattering.

**Figure 5:**
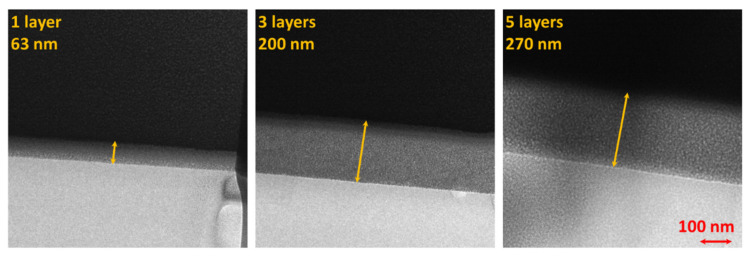
FIB produced cross-section TEM images of one, three, and five photocatalyst layers on PMMA substrate.

The SEM images of the TiO_2_ coating on the PMMA cross-section and surface can be seen in Figure 5. The coating thickness was determined using transmission electron microscopy’s focused ion beam (TEM-FIB) analysis. The coating cross-section showed that the sample thickness for one TiO_2_ coating was 63 nm, for three it was 200 nm, and for five layers, it was 270 nm. SEM and TEM-FIB showed that the produced coatings were homogenous and uniform in thickness. Similar coherence could be observed in SEM energy dispersion spectroscopy (EDS) (see Figure S3). Correlation between the deposited coating thickness, titanium concentration, and a number of layers can be seen in Supplementary Materials Figure S4.

**Figure 6:**
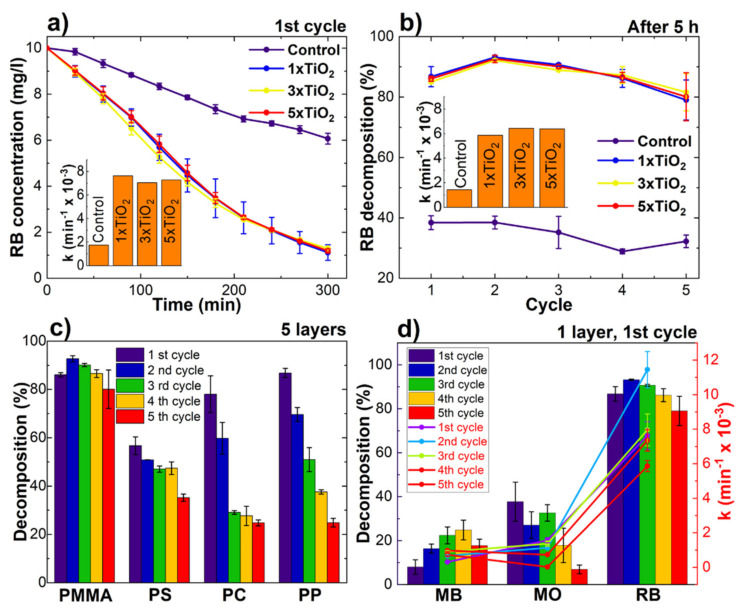
Photocatalytic activity measurements of TiO_2_ coatings on the PMMA substrate using RB during: (**a**) the first cycle; (**b**) summary during all five cycles; (**c**) comparison of PMMA and other polymers; (**d**) and comparison between RB and other organic dyes.

The photocatalytic degradation of rhodamine B is depicted in Figure 6. No significant differences in the degradation rate could be observed between PMMA samples with different numbers of photocatalyst layers (Figure 6a,b). For the uncoated PMMA samples, the amount of decomposed RB dye during 5 h of irradiation varied between 38.42% during the first cycle and 28.90% during the fourth cycle (Figure 6b). For the coated samples, the amount of decomposed dye was in the interval between 79.02% (one layer, fifth cycle) and 93.18% (one layer, second cycle). RB is not UV stable and can degrade via photolysis [18]. It was shown that the half-life of RB decomposition at room temperature, using a 250 W UV source, with the maximum absorption of 2 was 281 min [19]. In our case, the absorption value at spectra maximum on average was 2.1 a.u. and the light source was 100 W. This is a reason that the decomposition rate was lower for the control samples here than stated in the literature.

The TiO_2_ coatings on PMMA were stable during all five testing cycles. An insignificant decrease in the decomposed dye amount for TiO_2_ coatings on PMMA can be observed between the first and fifth cycle (7–5%) (Figure 6b). The photocatalytic activity evaluation using RB degradation of one TiO_2_ coating layer on PMMA, PS, PC, and PP can be seen in Figure 6c. The photocatalytic activity for coatings on other polymer substrates was lower and more unstable in comparison with PMMA. The lower photocatalytic activity and degradation rate can be related to inhomogeneous and loosely bonded coatings. In addition, the coatings on other polymer substrates were less stable when compared with coatings on PMMA. The lowest stability could be observed when the substrate material was used PP. This can be attributed to poor TiO_2_ nanoparticle adhesion to the substrate, as discussed above. For PC, a significant decrease in photocatalytic activity was observed during the first two cycles due to separation of loose TiO_2_ particles. After the second cycle, the coating became stable. More detailed information about photocatalytic activity using different organic dye solutions could be found in Supplementary Information (Figures S5–S7).

**Figure 7:**
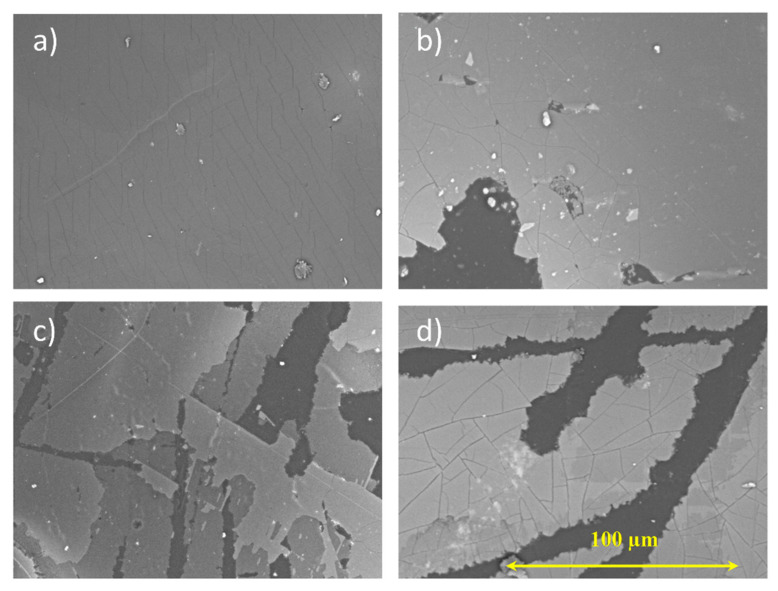
The SEM images for the TiO_2_ coatings containing three photocatalyst layers on (**a**) PMMA before and (**b**) after dye degradation with MB, (**c**) MO, and (**d**) RB.

Coating durability on PMMA was analysed based on a SEM image comparison before and after the photocatalysis tests in Figure 7. The SEM images showed a visible degradation in all coatings. Significant loss of the TiO_2_ coating could be observed for almost all samples after the photodegradation of organic dyes. The highest levels of degradation could be observed for samples that were studied using the degradation of methyl orange. The observed layer separation corresponded to a dramatic decrease in dye degradation speeds during repeated experiments using MO. The coating degradation for rhodamine B samples was less prevalent than in methyl orange. The RB photocatalyst losses corresponded to the level of degradation of dye reported in Figure 6. After the degradation of methylene blue, the photocatalyst layer degradation was less observable, which corresponded to the adsorption phenomena of MB, as previously stated. A similar trend could also be observed using optical microscopy (Figure S8).

**Figure 8:**
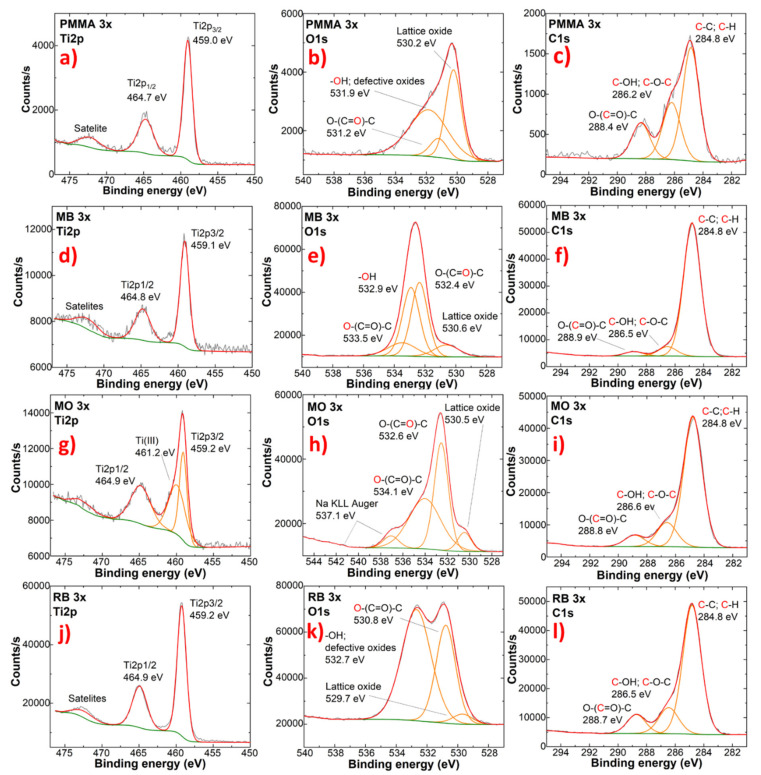
XPS measurements of high-definition scans of Ti 2p, O 1s, and C 1s spectra. (**a**–**c**) before photocatalysis (PMMA samples) and after photocatalysis using (**d**–**f**) MB; (**g**–**i**) MO and (**j**–**l**) RB, respectively.

The XPS spectra of the TiO_2_ coating before and after photocatalysis can be found in Figure 8. The Ti 2p spectra showed no significant changes in the TiO_2_ layer, and no formation of Ti^3+^ species, except after MO photocatalysis. The Ti 2p spectra showed the presence of Ti^3+^ species in samples with a different number of photocatalyst layers when used for MO degradation. The oxygen O 1s spectra showed characteristic TiO_2_ lattice oxygen in all of the measured samples with the TiO_2_ coating. The relative amount of lattice oxide decreased quite significantly, which can be explained by organic dye adsorption on the analysed sample surface and the exposure of PMMA substrate in the analysed area due to coating loss. In the case of methyl orange, the adsorption of Na can be detected, based on sodium Auger peaks. The XPS spectra of C 1s showed all functional groups of PMMA [20]. Differences in the individual peak intensities can be attributed to the TiO_2_ coating intermitter layer and surface contaminant formation. It is possible that adsorbed organic dyes give their signal in C 1s XPS spectra [21]. XPS spectra for all coatings on PMMA can be found in the Supplementary Materials (Figures S9–S12).

## 3. Discussion

Durable and photocatalytically active TiO_2_ nanoparticle coatings were obtained on various organic substrates at room temperature. Deposition is enabled by ultra-small TiO_2_ ligand-free nanoparticles with a size below 4 nm. Stripped TiO_2_ nanoparticles showed strong particle–particle interaction [22], which is responsible for the formation of dense coatings. This was confirmed by the SEM of cross-sections. In our approach, particles do not agglomerate, thus transparent coatings can be obtained on the PMMA substrate. The transparency also depends on the substrate material and solvent used. Substrates such as PC and PS cannot be applied for nanoparticle systems in DMF because porous non-transparent structures are formed. This could be related to the high solubility of the substrate material and precipitation by solvent exchange with air humidity.

TiO_2_ coatings showed photocatalytic activity for the degradation of various organic substances under UV irradiation, as expected. Overall, the coating showed high photocatalytic activity, thanks to small particle size and their crystalline anatase structure. Our synthesised TiO_2_ nanoparticles showed much higher photocatalytic activity in comparison with the commercially available Aeroxide^®®^ P25 (Figure 2). The high photocatalytic activity can be related to the high charge mobility of the anatase phase and small particle size comparable to the photoinduced charge carrier diffusion length 120–1000 nm [23]. High photocatalytic activity can be explained with small particle size, which was 5–6 times smaller than P25. It is well-reported that the particle size is a crucial factor in the rates of electron-hole recombination [24]. It has been repeatedly demonstrated that the smaller particles showed faster degradation kinetics [25]. Charge had aa shorter distance to reach the surface for catalytic reactions, thus decreasing the probability of recombination.

The photocatalytic activities for the TiO_2_ coatings obtained by different methods are compared in Table 1. However, it is hard to compare the photocatalytic activity from one work to another due to different experimental conditions. Considering the sample area and dye concentration, the rate constants for dye degradation at our work were relatively high. Our samples were in between the most active among the coatings listed in Table 1.

Photocatalytic TiO_2_ coatings on PMMA showed good stability over five dye degradation cycles. However, some delamination from the substrate could be observed from cycle to cycle. This can be attributed to the photocatalytic degradation of the substrate material. This could be eliminated by introducing an inert intermediate layer such as SiO_2_ and will be addressed in future studies.

## 4. Materials and Methods

### 4.1. Synthesis of TiO_2_ Nanoparticles

TiO_2_ nanoparticle synthesis is based on the modified version presented by Emmanuel Scolan and Clement Sanchez [37] where 9.05 mL of titanium tetra n-butoxide (97%, Sigma-Aldrich, St. Louis, MO, USA) is added dropwise to the mixture containing 12.358 mL n-butanol (≥99.5%, Merck, stored over CaH_2_, Germany) and 8.268 mL of acetylacetone (≥99%, Merck, China). After the addition of the precursor, the reaction mixture was brought to the boiling point. A preheated mixture of 4.865 g of deionised water and 1.76 g of 4-dodecylbenzene sulfonic acid (4-DDBSA) (≥95%, Sigma-Aldrich, Germany) was added to the reaction mixture dropwise. After the addition of 4-DDBSA, the solution was refluxed overnight. The next day, the solution was cooled, and the formation of yellowish particles was observed. The precipitate was washed twice with methanol (gradient grade for liquid chromatography, Supelco, Germany) and centrifuged (2-16P, Sigma, Germany) at 2000 g for 1 h. After the last washing, particles were allowed to separate at 4000 g for 1 h. After the washing, particles were dispersed in N,N dimethylformamide (DMF) (99%, Merck, Poland) with a concentration of 100 g/l [38].

Before the deposition of films, the surfactant was removed from the particles. A total of 39 mL of hexane (≥97%, Merck, Israel) was added to the 13 mL of TiO_2_ colloid in DMF and 13 mL of triethyloxonium tetrafluoroborate (EtO_3_BF_4_) (≥97%, Sigma-Aldrich, Switzerland) solution in dichloromethane (DCM) (for analysis, Supelco, Germany) (20 mg/mL) was added dropwise, followed by 20 mL of toluene (≥99.7%, Merck, Israel). The solution was mixed for a short amount of time and allowed to separate. As previously stated, the large volume of solution was decanted, and the precipitate was washed with methanol. After washing cycles, solids were dispersed in DMF with a concentration of 100 g/l. The solution was used immediately after preparation [39].

### 4.2. Coating Deposition

Commercially available polymer samples with dimensions of 25 × 25 × 4 mm were used for all experiments. Before the deposition, polymer substrates were washed with ethanol and treated using plasma (PDC-002-CE, Harrick Plasma, Ithaca NY, USA, setting: high) for 15 min. Coatings were fabricated using a custom-made spin coater. Coatings were deposited at 1500 rpm for 30 s, allowing the DMF to evaporate between layers. Samples containing one, three, and five layers were produced. The solvent was chosen due to its ability to partially dissolve the studied polymers (except PP) to improve the adhesion of the photocatalytically active layers on the substrate.

### 4.3. Sample Characterisation

The particles were characterised using Raman spectroscopy (inVita, Renishaw, Wotton-under-Edge, UK), XRD (X’Pert, PANalytical, Malvern, United Kingdom), and TEM (Tecnai G20, FEI, Waltham, MA, USA) imaging at 200 kV. The samples for TEM analysis were placed on a perforated carbon film on a 400-mesh copper grid (S147-4, Agar Scientific, Stansted, UK). The particle size distribution was determined using ImageJ 1.53K software [40]. Band gap energy was determined by DRS measurements (SolidSpec-3700, Shimadzu, Japan), Absorption data were calculated by the Kubelka–Munk theory from the reflectance data. Coatings were examined using XPS (ESCALAB Xi+, Thermo Scientific, Waltham, MA, USA) before and after the photocatalysis tests. The residual charging on the sample surface was compensated by using automatic built-in charge compensation tools [41]. Experimental data were fitted using Avantage 5 software using an advantageous carbon peak at 284.8 eV as a calibration point [42]. Coatings before and after the photocatalysis tests were studied using an optical microscope (DM LP, Leica with Leica DC digital camera, Wetzlar, Germany), SEM (TM 3000, Hitachi, Tsukuba, Japan), and EDS. Coating thickness was determined using SEM-FIB produced lamellas (Lyra, Tescan SEM with micromanipulator, 30 kV current, STEM 3+ detector, Czech Republic) in TEM. The surface contact angle was measured using deionised water and diiodomethane (stabilised by copper, Sigma-Aldrich, India) to calculate the surface free energy (SFE) values (Drop shape analyser, Kruss, Hamburg, Germany).

### 4.4. Photocatalysis Test

Photocatalytic activity of the TiO_2_ coatings was estimated following a modified version of the ISO 10678:2010 standard [43] by the degradation of organic dyes such as methylene blue (Sigma-Aldrich, India), methyl orange (Sigma-Aldrich, India), and rhodamine B (≥95% for HPLC, Sigma-Aldrich, St. Louis, MO, USA) in deionised water with a concentration of 10 mg/L. A glass cylinder, with an inside diameter of 20 mm, was placed on the coating surface and fixed in place using silicon oil (DC 200, Fluka, Switzerland). Silicon oil is needed to avoid the outflow of dye solution and ease the removal of cylinders after the test. During the experiment, 2 mL of dye solution was added to the glass cylinders and covered with a glass plate. A schematic depiction of the photocatalysis measurement setup is shown in Supplementary Figure S13 [44,45]. Before the irradiation, the samples were left in a dark dye solution for 12 h at room temperature to achieve an adsorption–desorption equilibrium of dye on the surface. After adsorption, the dye solution was replaced with the fresh one. Samples were irradiated in a dark box with a UV light source (Black-Ray^®®^ B-100AP, UVP, 365 nm, 100 W mercury spot lamp, Upland, CA, USA) 20 cm above the sample surface. Samples were stirred every 30 min by using a single-use pipette. Sample of a 1.5 mL was taken and transferred to a PMMA semi-micro cuvette for the measurement of absorbance spectra using a UV–Vis spectrophotometer (Genesys 10S, Thermo Scientific, China). The dye decomposition was calculated by following the decrease in the intensity of each dye’s maximum absorbance values at 664, 465, and 554 nm for methylene blue, methyl orange, and rhodamine B, respectively. The measured absorption spectra for different dye solutions are demonstrated in Supplementary Figure S14. After measuring the absorption spectra, the dye solution was transferred back to the glass cylinder [46]. The measurement was repeated at certain time intervals for a final test duration of 5 h. The dye concentration was calculated based on a calibration graph that was recalibrated before every measurement [46].

The photocatalytic activity of laboratory made TiO_2_ nanoparticles were compared to the commercially available TiO_2_ nanopowders (Acros Organics, Aeroxide ^®®^ P25 with an average particle size of 25 nm, Belgium). A total of 0.8 mg/mL was dispersed in MB solution (5 ppm) using sonification. The suspension was placed under UV light with vigorous stirring. A 1.5 mL sample was collected every 10 min and centrifuged for 5 min to avoid nanoparticle effects on the UV–Vis measurements. Samples were analysed over a 60 min period.

The dye decomposition was calculated from the concentration change [47] following Equation (1) [47,48]:

(1) $$\mathrm{Decomposition}\;\left(\%\right)=\frac{C_{0}-C_{t}}{C_{0}}\cdot 100\%$$

where C_0_ is the initial concentration of used dye and C_t_ is the dye concentration at time “t”.

The kinetics of dye decomposition can be described as a pseudo-first-order kinetic reaction. The pseudo-first-order reactions can be expressed in Equations (2) and (3) [49]:

(2) $$\frac{d\left[C\right]}{\mathrm{dt}}=-K\left[C\right]$$

(3) $$\ln\left(\frac{C_{0}}{C}\right)=\mathrm{Kt}$$

where K is the kinetical constant of the pseudo-first-order reaction (min^−1^), C0 is the initial concentration of organic dye, and C is a specific concentration in given time t (min). Kinetic constant K was determined by the ln(C_0_/C) vs. t plot. Since the R2 value for most of the plots was higher than 0.95, the experimental data can be attributed to the pseudo-first-order kinetic reaction model [23].

For a better understanding of different dye photochemical degradation, the relative decomposition in comparison with the uncoated control samples was calculated:

(4) $$\mathrm{Relative}\;\mathrm{decomposition}\;\left(\%\right)=\frac{C_{\mathrm{control}}-C}{C_{\mathrm{control}}}\cdot 100\%$$

## 5. Conclusions

In this work, we demonstrated how it is possible to obtain durable and optically clear TiO_2_ coatings on PMMA by using ultra-small TiO_2_ nanoparticles. TiO_2_ coatings on PMMA demonstrated 97% light transmittance compared with the uncoated substrate without significant surface damage. The produced coatings demonstrate good photocatalytic properties in UV light with repeatable results. The RB degradation rate dropped 5–7 % between the first and fifth cycle. It is possible to obtain TiO_2_ coatings on different polymers but the interaction of DMF and the polymer surface creates defects, which lead to the loss of transparency and poorer photocatalytic degradation rates. A simple and easy method was demonstrated for obtaining TiO_2_ coatings on a PMMA substrate with enhanced photocatalytic properties.

## Figures and Tables

**Table 1:** Comparison of the different photocatalytic coatings found in the literature.

Substrate	Coating Method and Temperature	Area	Dye	Concentration of the Dye	Light Source and Intensity	Rate Constant	Ref.
Glass	Dip-coating and annealing at 500 °C	10 cm^2^	MB	30 µM	365 nm UV lamp with 3.48 mW/cm^2^	k = 1.5 × 10^−2^×min^−1^	[26]
Glass	Dip-coating and annealing at 600 °C	6.5 cm^2^	MB	100 mg/L	370 nm 15 W UV light	k = 2.6 × 10^−3^ min^−1^	[27]
Glass	Atomic layer deposition at 250 °C and 350 °C	23 cm^2^	MB	1 mM	365 nm UV lamp at distance of 11 cm	k = 4.6 × 10^−3^ min^−1^	[28]
Quartz Glass	Dip-coating and annealing at 120 °C	18.75 cm^2^	RB	10 µM	Four 4 W UV lamps (365 nm)	k = 1.9 × 10^−3^ min^−1^	[29]
Al_2_O_3_ membrane	Dip-coating and annealing at 500 °C	4.15 cm^2^	RB	25 µM	40 W/m^2^ UV lamp	1007 mg × m^−2^ × h^−1^	[30]
Glass and PC	Magnetron sputtering	18.75 cm^2^	RB	0.5 mg/L	Hg tube lamp with a wavelength of 254 nm at 11 cm distance	k = 3.5 × 10^−3^ min^−1^ for polycarbonatek = 2.9 × 10^−3^ min^−1^ for glass sample	[31]
Pyrex spheres	Dip-coating and annealing at 450 °C	0.58 cm^2^ per sphere.	MB	5 mg/L	Visible light 32 mW/cm^2^ and UV light 35 mW/cm^2^	For MB k = 4.6 × 10^−3^ min^−1^ (UV) k = 3.4 × 10^−3^ min^−1^ (Vis)	[32]
Activated carbon fibres	Molecular adsorption-desorption	49.5 cm^2^	MB	2.498 mmol/L	24 W mercury lamp (254 nm) at a distance of 12 mm	k = 3.1 × 10^−2^ min^−1^	[33]
Glass	Dip-coating	10 cm^2^	MB	25 µM	He-Cd laser (442 nm) at a distance of 6 cm	k = 4.2 × 10^−3^ min^−1^	[34]
PC	Dip-coating	8.75 cm^2^	MB	5 µM	4W UV lamp (254 nm) 11 mW/cm^2^	k = 2.5 × 10^−2^ min^−1^	[35]
Glass	DC magnetron sputtering at 200 °C	1.75 cm^2^	RB	1 µM	200W Hg lamp (280–380 nm) at a distance of 12 cm	k = 7.8 × 10^−3^ min^−1^	[36]
PC, PMMA, PS, and PP	Spin-coating	3.14 cm^2^	RB	10 mg/L	100W Hg lamp (365 nm) at a distance of 20 cm	k = 1.1 × 10^−2^ min^−1^	This work

## Data Availability

Not applicable.

## Supplementary Materials

The following supporting information can be downloaded at: https://www.mdpi.com/article/10.3390/ijms232112936/s1.
