# Supplementary material for: Photocatalytic Activity of TiO2 Coatings Obtained at Room Temperature on a Polymethyl Methacrylate Substrate
Source: Int J Mol Sci. 2022 Oct 26;23(21):12936. doi: 10.3390/ijms232112936 (PMC9655194; doi:10.3390/ijms232112936)
Supplement: Supplementary file 1 [file ijms-23-12936-s001.zip › ijms-1977453-supplementary.pdf]

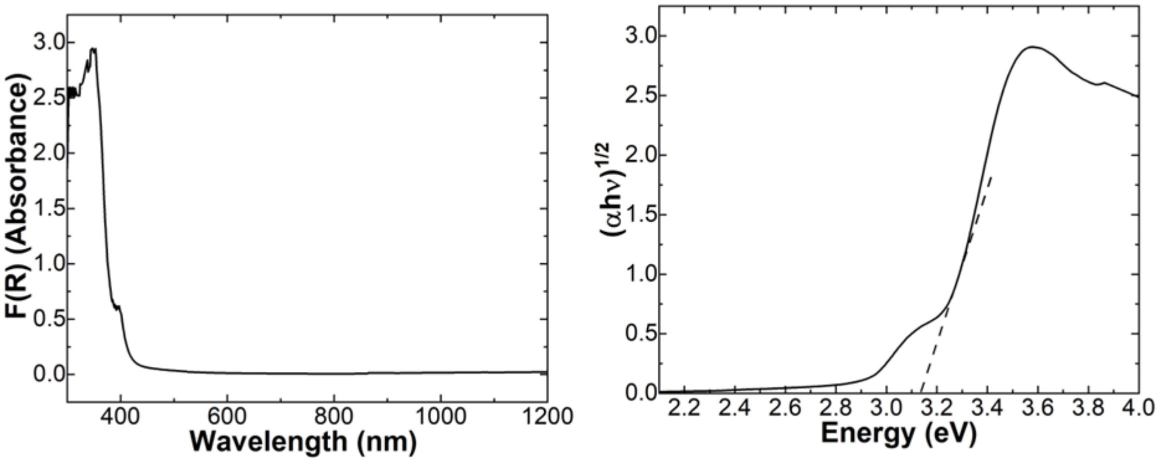

Figure S1. DRS spectra for synthesized nanoparticles and corresponding Tauc plot.

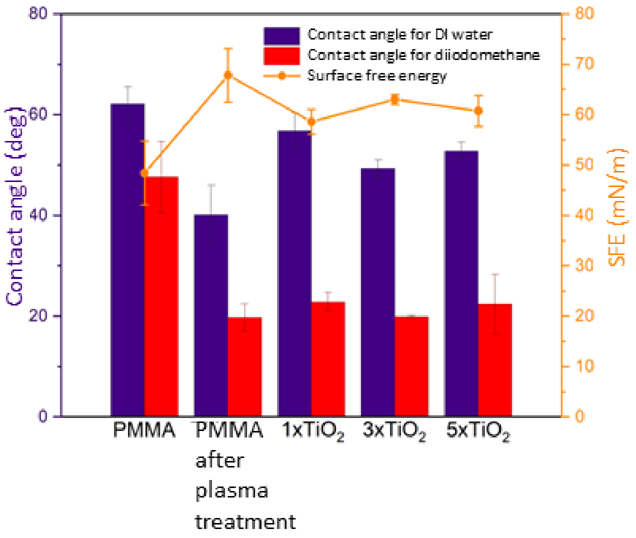

Figure S2. Contact angle and surface free energy (SFE) values for PMMA substrates before and after deposition of TiO_2 coating.

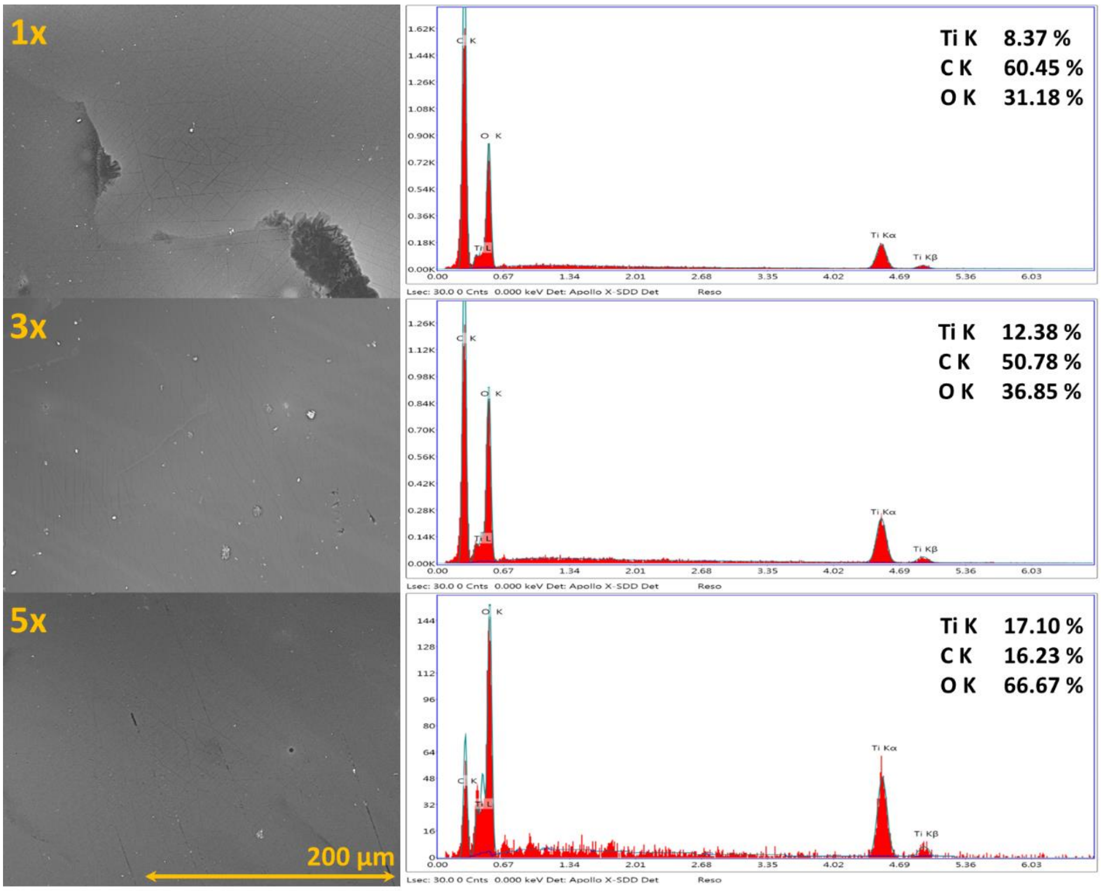

Figure. S3. SEM-EDS analysis of TiO_2 coatings on PMMA substrate.

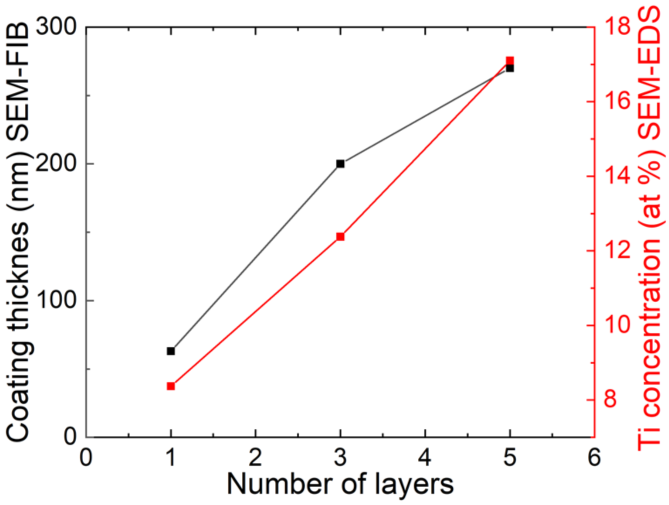

Figure S4. Correlation between number of layers, coating thickness and Ti concentration.

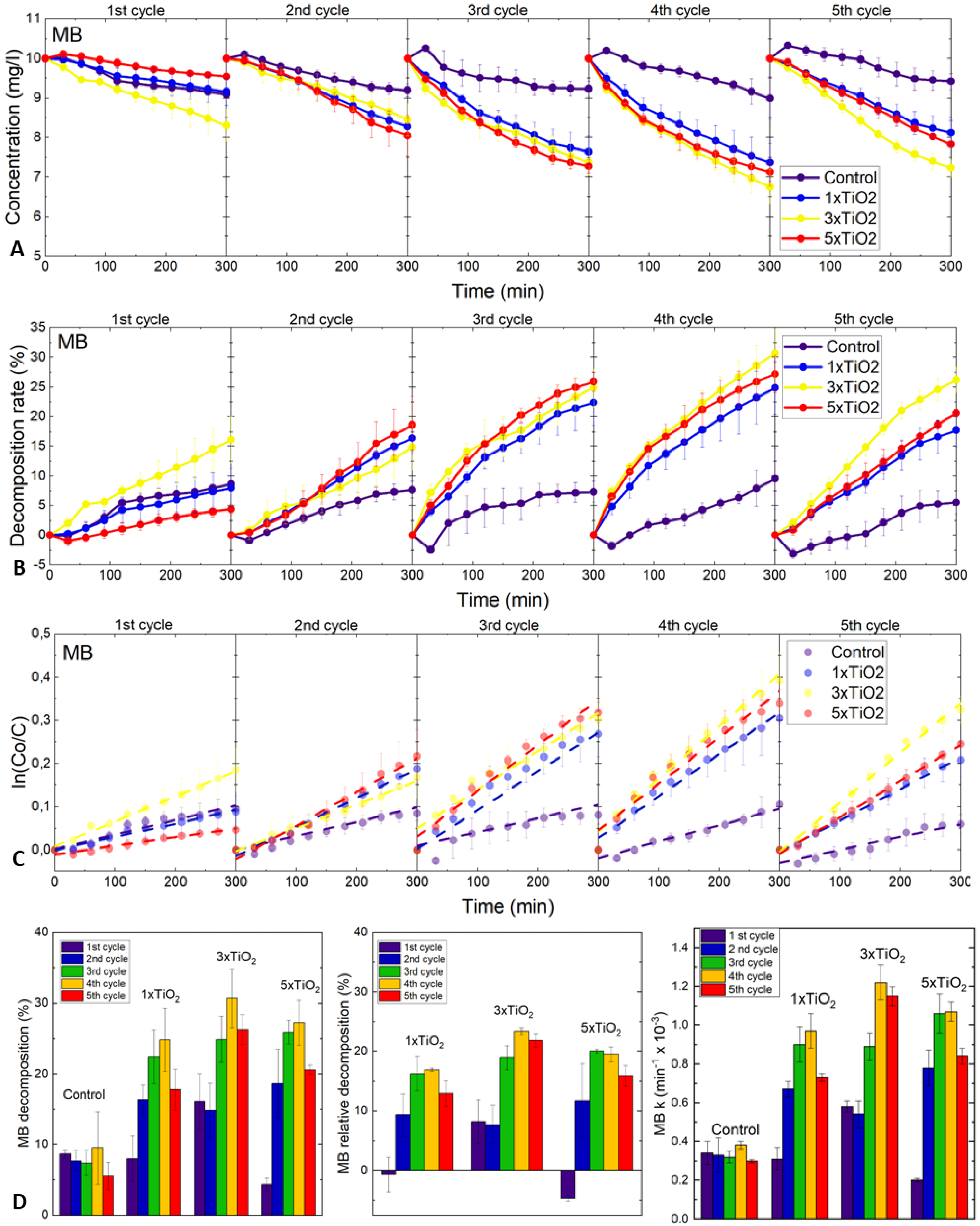

Figure S5. Photocatalytic degradation of methylene blue. Concentration changes (A), decomposition rate (B) and kinetic information (C). MB decomposition (%) and MB relative decomposition (%) after 5 hours and decomposition kinetic constant (min^{-1} x 10^{-3}) (D).

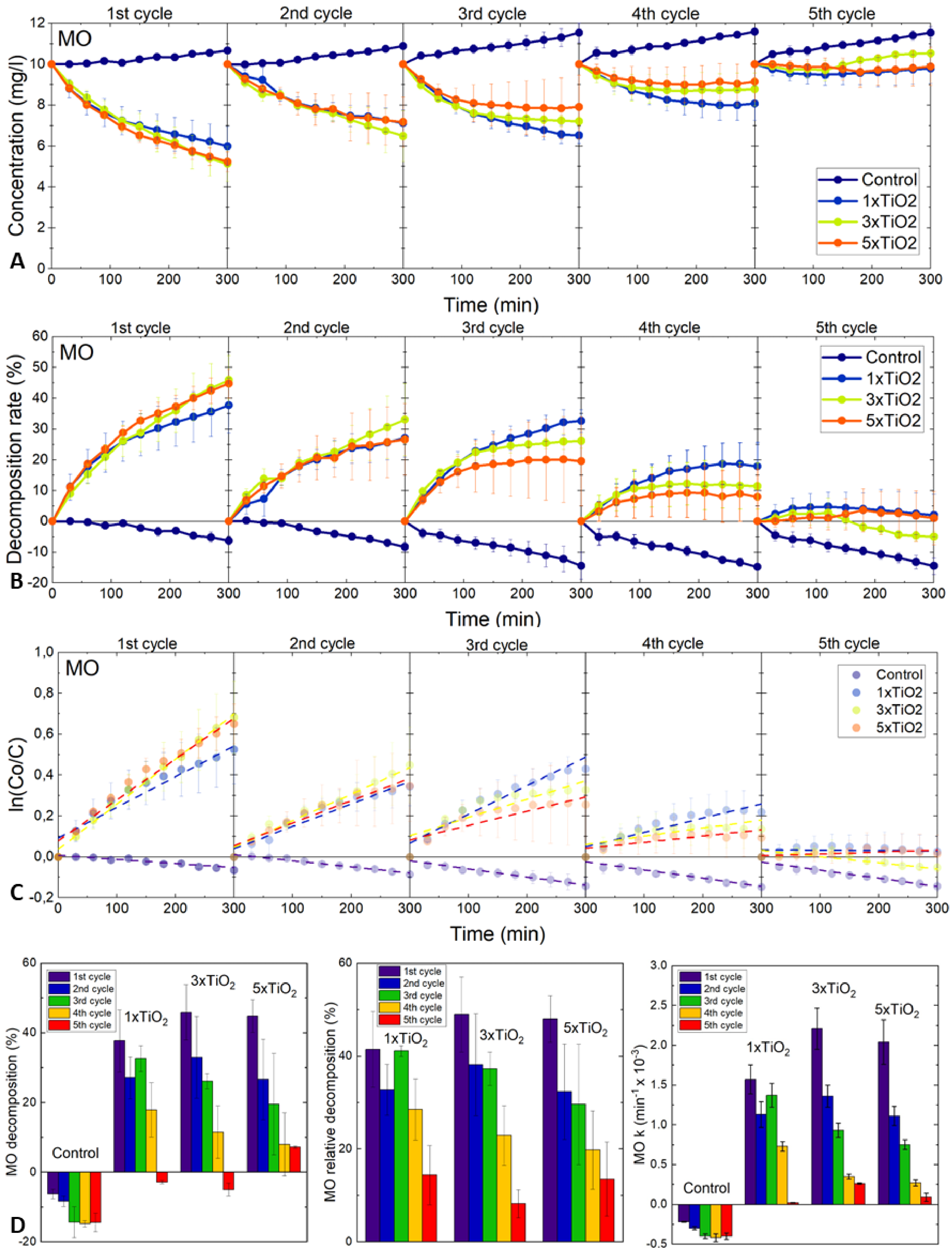

Figure S6. Photocatalytic degradation of methyl orange. Concentration changes (A), decomposition rate (B) and kinetic information (C). MO decomposition (%) and MO relative decomposition (%) after 5 hours and decomposition kinetic constant (min^{-1} x 10^{-3}) (D).

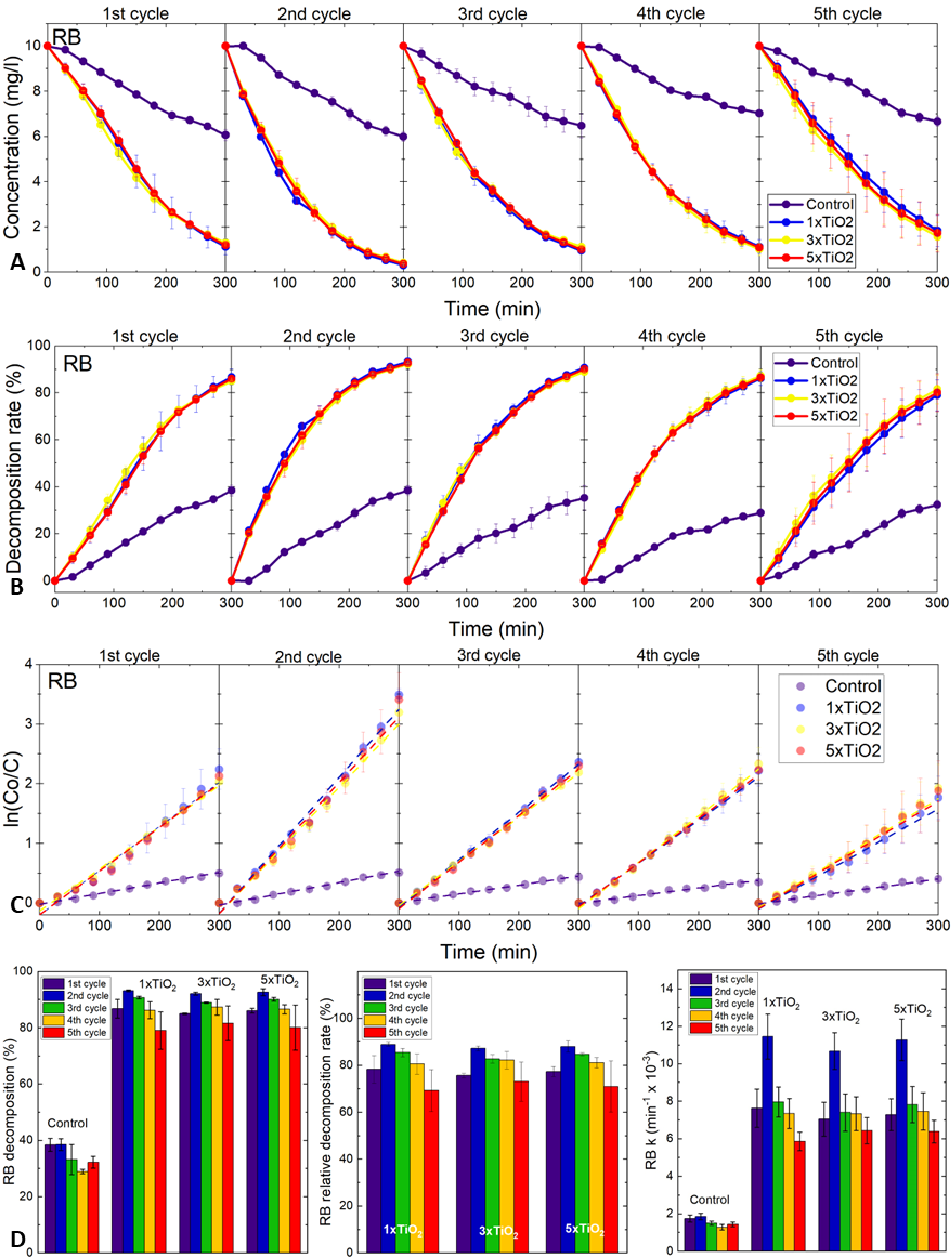

Figure S7. Photocatalytic degradation of rhodamine B. Concentration changes (A), decomposition rate (B) and kinetic information (C). RB decomposition (%) after 5 and RB relative decomposition (%) after 5 hours and decomposition kinetic constant (min^{-1} x 10^{-3}) (D).

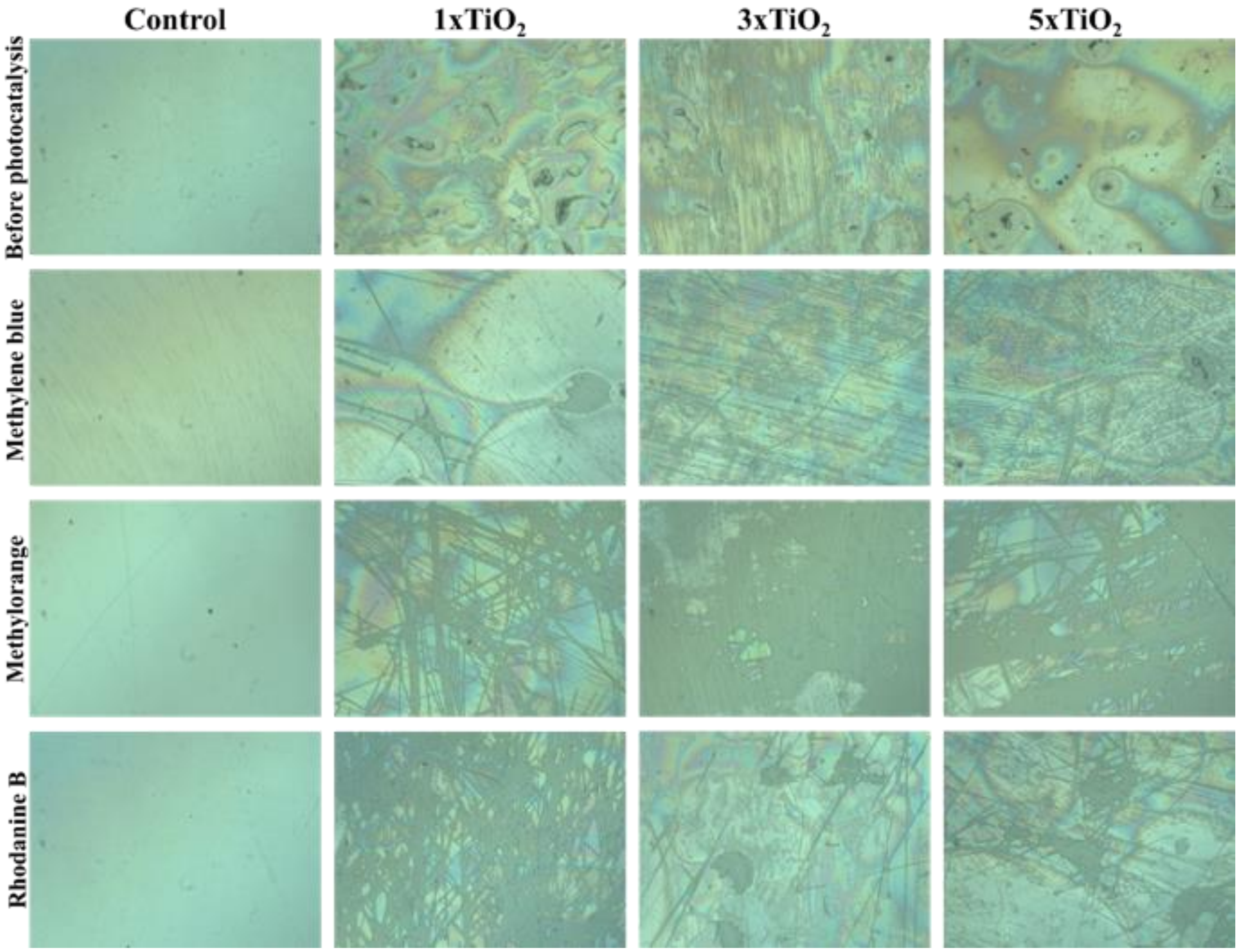

Figure S8. Microscopy photography at 4x optical zoom before and after photocatalysis measurements.

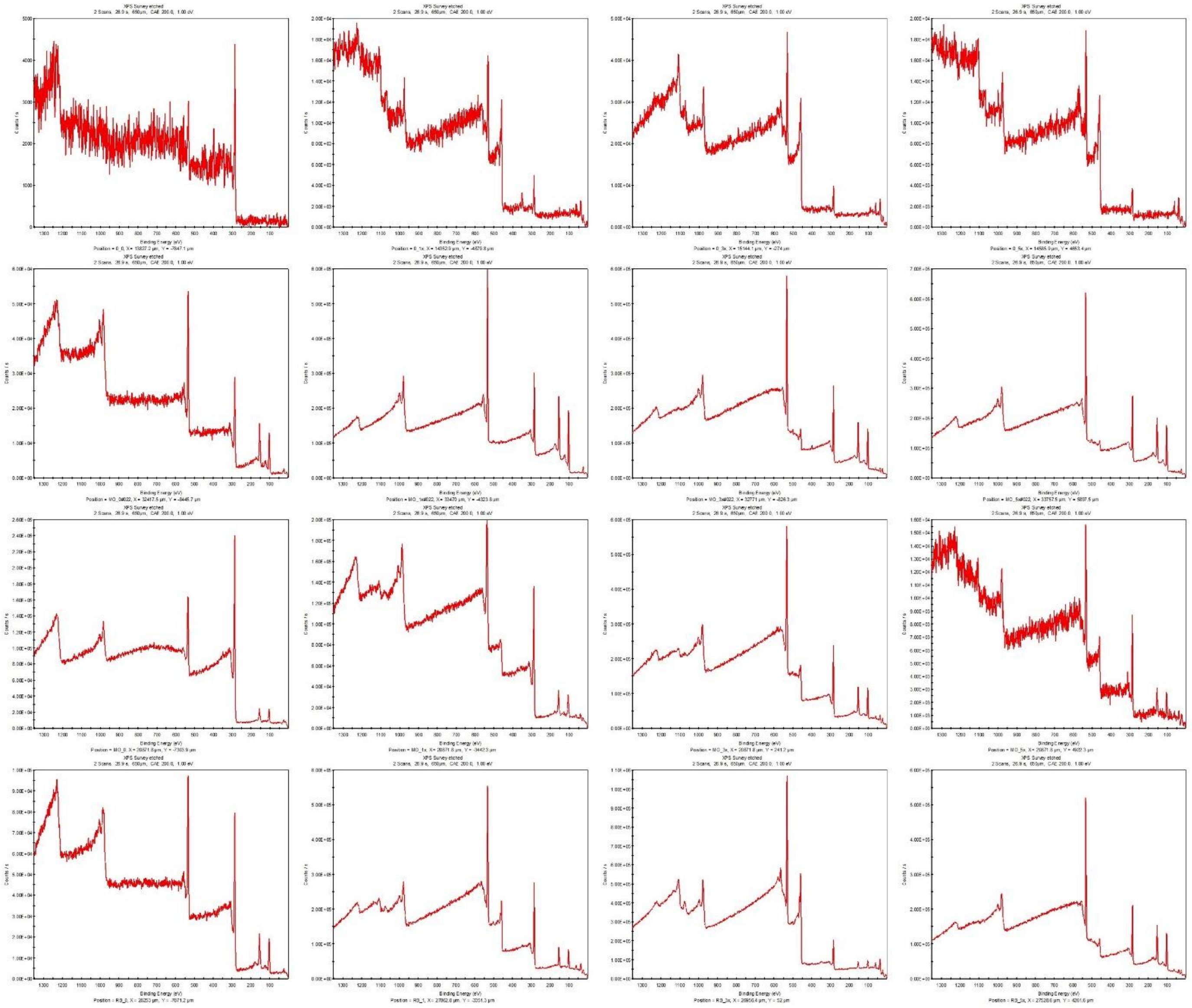

Figure S9. XPS survey spectra of TiO_2 coatings on PMMA before and after photocatalysis tests.

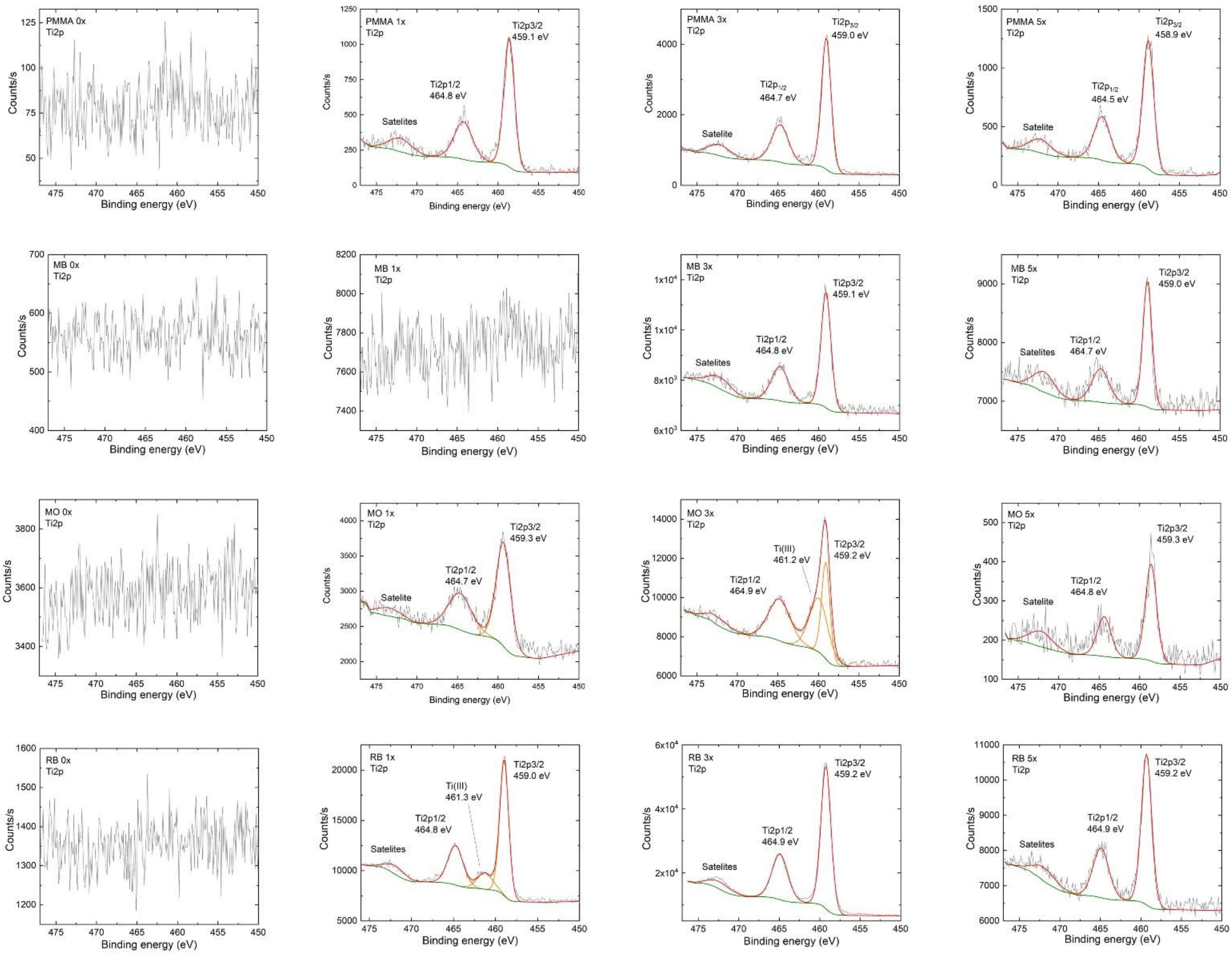

Figure S10. Ti 2p XPS spectra of TiO_2 coatings on PMMA before and after photocatalysis tests.

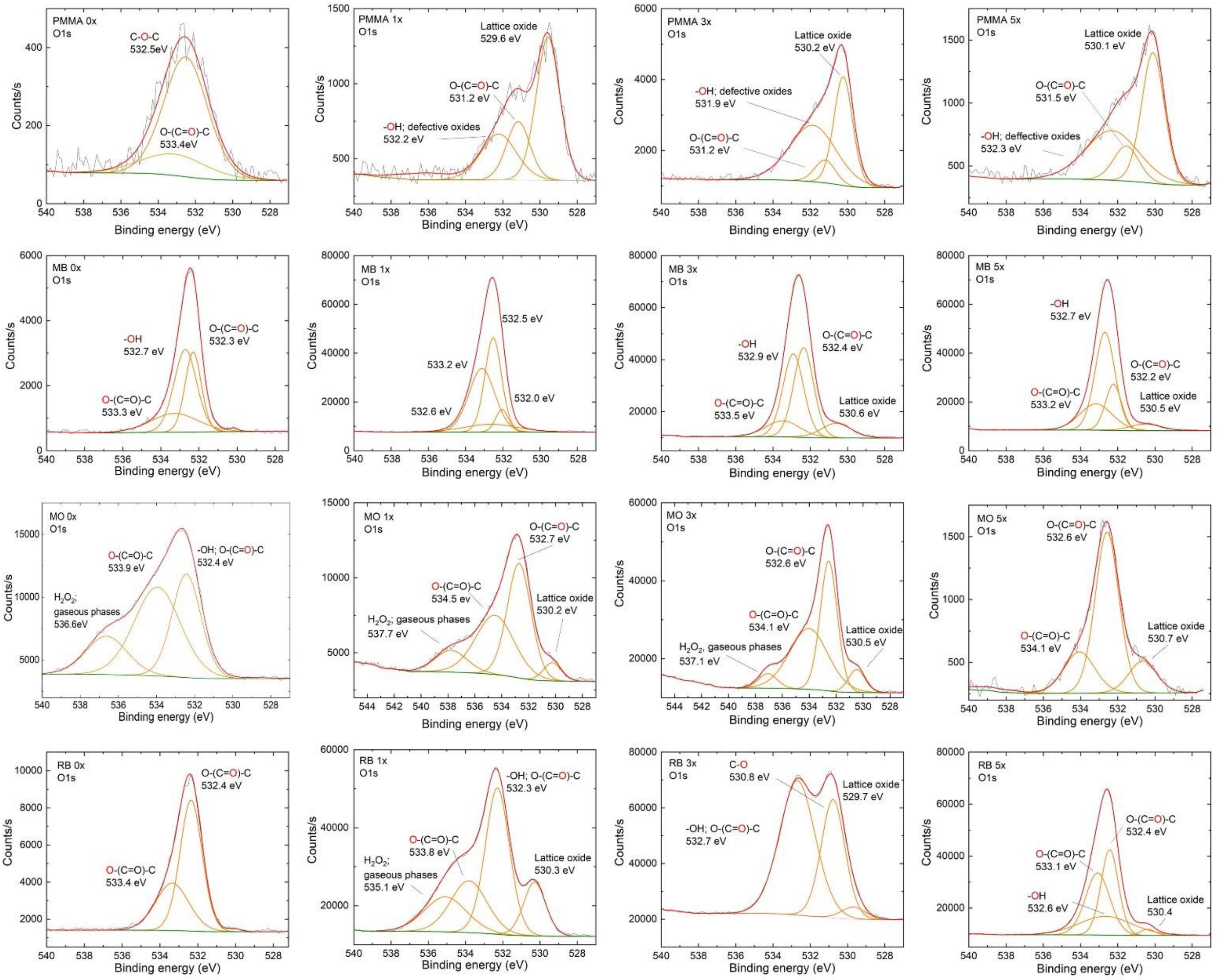

Figure S11. O 1s XPS spectra of TiO_2 coatings on PMMA before and after photocatalysis tests.

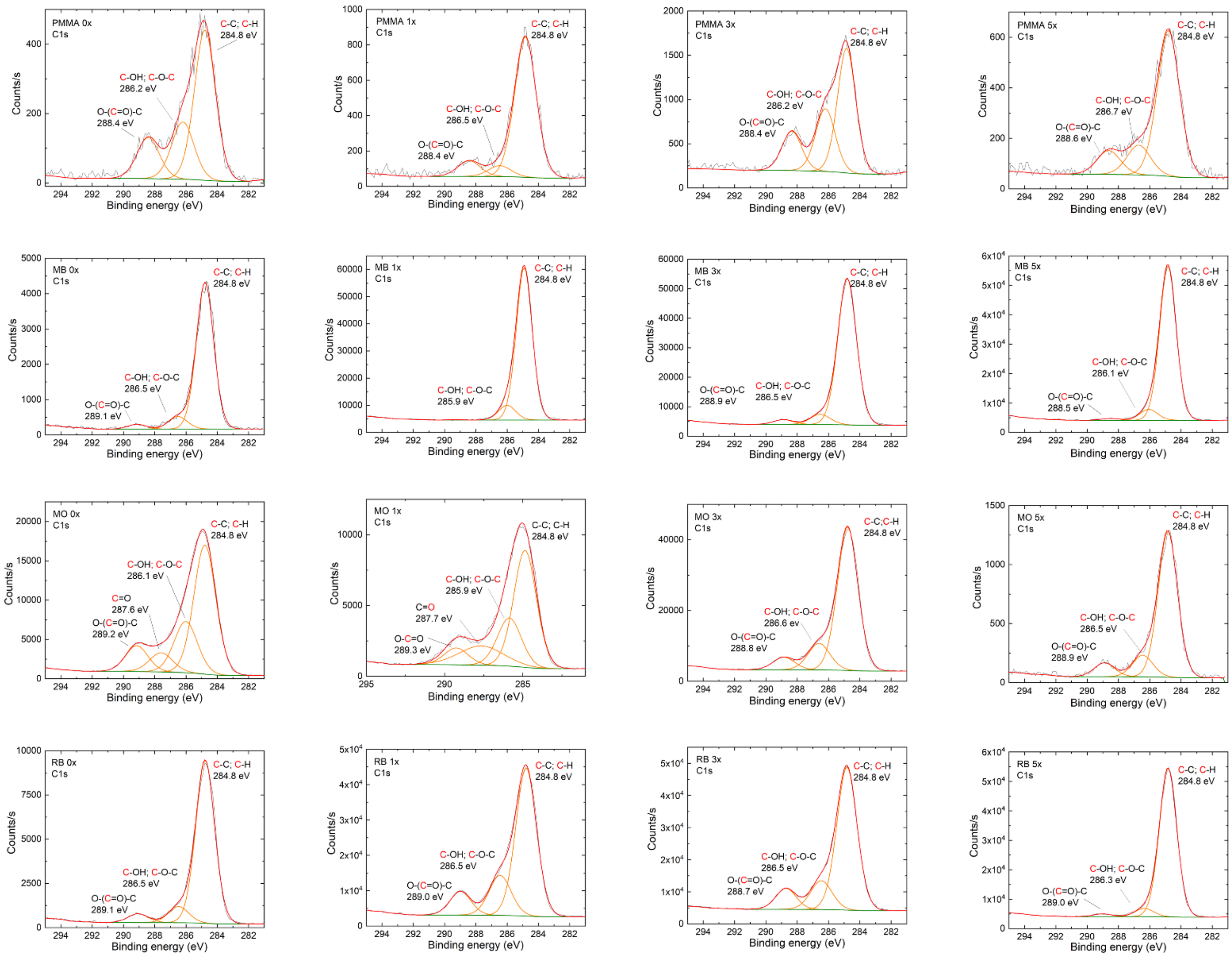

Figure S12. C 1s XPS spectra of TiO_2 coatings on PMMA before and after photocatalysis tests.

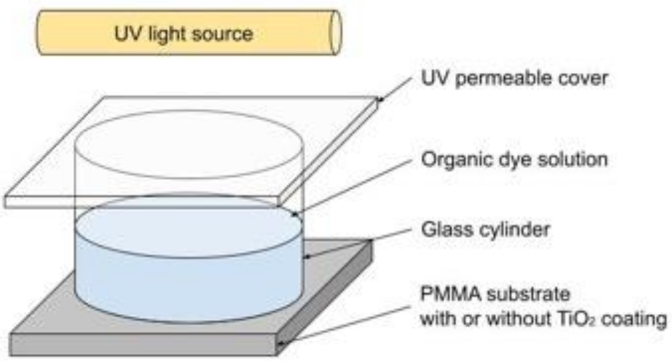

Figure S13. Schematic depiction of the photocatalysis measurement set-up.

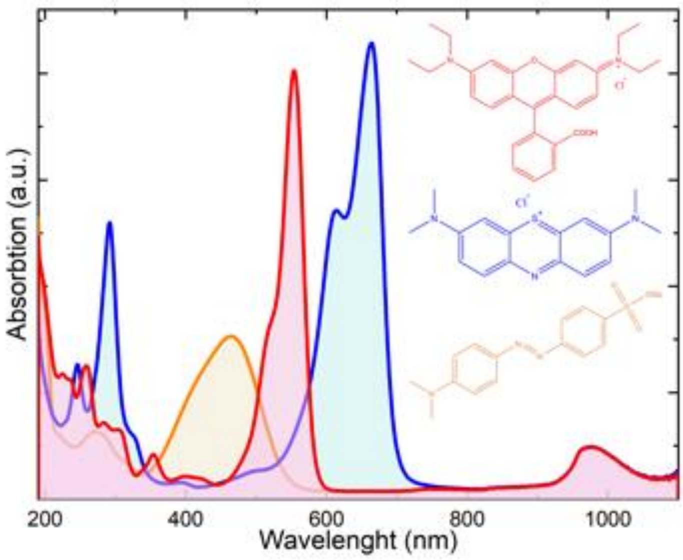

Figure S14. UV-VIS spectra of rhodamine B (red), methylene blue (blue) and methyl orange (orange).
